# When Does Physician Use of AI Increase Liability?

**DOI:** 10.2967/jnumed.120.256032

**Published:** 2021-01

**Authors:** Kevin Tobia, Aileen Nielsen, Alexander Stremitzer

**Affiliations:** 1 Georgetown University Law Center, Washington, DC; and; 2 Center for Law and Economics, ETH Zürich, Zürich, Switzerland

**Keywords:** artificial intelligence, liability, precision medicine

## Abstract

An increasing number of automated and artificial intelligence (AI) systems make medical treatment recommendations, including personalized recommendations, which can deviate from standard care. Legal scholars argue that following such nonstandard treatment recommendations will increase liability in medical malpractice, undermining the use of potentially beneficial medical AI.  However, such liability depends in part on lay judgments by jurors: when physicians use AI systems, in which circumstances would jurors hold physicians liable? **Methods:** To determine potential jurors’ judgments of liability, we conducted an online experimental study of a nationally representative sample of 2,000 U.S. adults. Each participant read 1 of 4 scenarios in which an AI system provides a treatment recommendation to a physician. The scenarios varied the AI recommendation (standard or nonstandard care) and the physician’s decision (to accept or reject that recommendation). Subsequently, the physician’s decision caused harm. Participants then assessed the physician’s liability. **Results:** Our results indicate that physicians who receive advice from an AI system to provide standard care can reduce the risk of liability by accepting, rather than rejecting, that advice, all else being equal. However, when an AI system recommends nonstandard care, there is no similar shielding effect of rejecting that advice and so providing standard care. **Conclusion:** The tort law system is unlikely to undermine the use of AI precision medicine tools and may even encourage the use of these tools.

See an invited perspective on this article on page 15.

Imagine that a woman has recently been diagnosed with ovarian cancer. To help determine the dosage of a chemotherapy drug, the treating hospital has adopted routine use of an artificial intelligence (AI) precision medicine tool. The AI tool advises, on the basis of the patient’s file, that a nonstandard dosage is most likely to succeed. But what if something goes wrong as a result of the treatment? Will the physician be judged harshly for accepting unorthodox treatment advice from a computer? Or might the physician be judged even more harshly for rejecting advice from a state-of-the-art tool?

The woman’s story is a hypothetical example from an important recent paper on AI in medicine (1). But this story may not remain hypothetical for long. Recent advances in AI medical technology make possible a wide range of personalized medical recommendation tools, some of which have achieved regulatory approval and are increasingly being adopted by medical providers (2).

However, despite the promise of these AI medical systems to improve patient outcomes, legal scholars have cautioned that tort law may create a substantial legal barrier to physicians’ uptake of AI recommendations: accepting certain AI recommendations may increase physicians’ risk of liability in medical malpractice (1). In particular, given tort law’s privileging of standard care, physicians who accept a personalized AI recommendation to provide nonstandard care would increase their risk of medical malpractice liability.

The purpose of this investigation was to contribute empiric evidence bearing on these questions: in which circumstances are physicians using AI systems more likely to be found liable, and how can physicians reduce their potential liability?

Traditionally, the answer to this question depends on custom. A physician must “exercise the skill and knowledge normally possessed” by other physicians (3). This customary standard is normally supported by expert witness testimony, clarifying the local or national practice. Often, jurors evaluate what the normal or average physician would do in light of conflicting testimony from dueling medical experts (4). Recently, some jurisdictions have moved to a “reasonable physician” standard (5). To help clarify jury decision making in both of these contexts, we study lay judgments about the “reasonable physician” in response to typical AI use cases. We also study how judgments about a “reasonable physician” relate to judgments about an “average physician.”

Of course, only a fraction of medical malpractice lawsuits reach a jury—many more settle (6). But even parties who ultimately settle their medical malpractice claims benefit from knowledge about the likely jury outcome if trial had ensued. For those, the results here provide evidence about the shadow of the law; the likely outcome of the court proceedings is an important input into settlement negotiations.

## MATERIALS AND METHODS

### Study Population, Design, and Setting

The study was conducted in March 2020. Participants were recruited from the United States through a Lucid survey and consisted of a nationally representative sample of 2,000 individuals stratified by age, race, and sex. Our sample size was based on a power analysis using G*Power. We ran power analyses to assess each of the preregistered tests, with a power of 0.95, to detect small effects (Cohen f = 0.10). The calculations indicated that a sample of 1,300 would be sufficient to assess each of the preregistered tests at this level. We anticipated excluding up to 30% of participants (e.g., for failing comprehension-check questions) and thus recruited 2,000 participants.

Participants were presented with different versions of a medical AI scenario. To minimize researcher degrees of freedom, the scenarios closely followed those of Price et al. (1), who introduced these vignettes without knowledge of the hypothesis of this project. In each scenario, a physician reviewed an ovarian cancer patient’s case file, which included routine input from a medical AI system called Oncology-AI. Participants were told that the AI system had all of the relevant regulatory approvals and provided a chemotherapy drug dosage recommendation.

**FIGURE 1.**
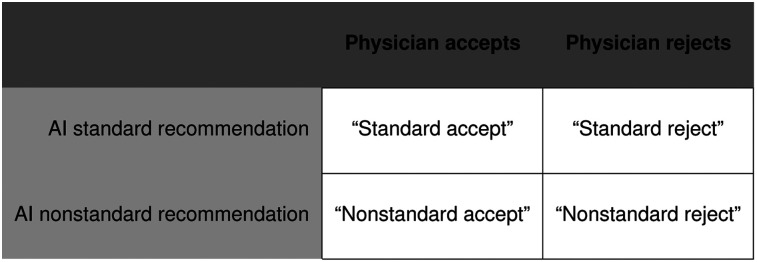
Experimental design that crosses recommendation (standard, nonstandard) with decision (accept, reject).

The study had a 2 × 2 between-subjects factorial design, varying the AI recommendation (recommending standard or nonstandard treatment) and the physician’s decision (accepting or rejecting the AI recommendation). Thus, participants were presented with 1 of 4 possible scenarios: a physician receives from an AI medical system either a standard treatment recommendation (900 mg every 3 wk) or a nonstandard treatment recommendation (4,500 mg every 3 wk). Then, the physician either accepts the recommendation or rejects it (Fig. 1). In all scenarios, the treatment choice causes harm, and participants evaluate these facts according to the same legal standard: was the decision one that could have been made by a reasonable physician?

**FIGURE 2.**
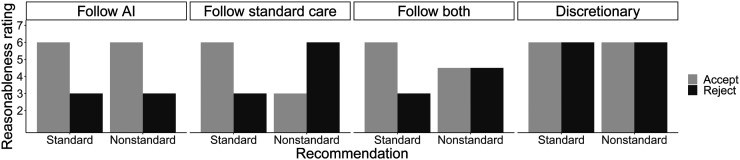
Experimental predictions of 4 models.

The study was designed to adjudicate among 4 plausible models of lay judgment of legal liability, with each model resulting from a different combination of 2 factors: provision of standard care and adherence to the AI recommendation. These 4 models make very different predictions about the pattern of results across the 4 scenarios and what a physician should do to minimize liability (Fig. 2).

The first model is to follow AI recommendations, as lay jurors are more inclined to hold physicians liable for rejecting AI recommendations. Some legal scholars have suggested that such a model is likely in the future as the use of AI precision medicine grows (1,7). The second model is to follow standard care, as lay jurors are more inclined to hold physicians liable for providing nonstandard care, regardless of the AI recommendation. This model reflects the presentation of current tort law by Price et al (1). The third model is to follow both; that is, lay judgment is affected by both factors: AI recommendation and standard care. This model predicts a significant interaction between recommendation and decision and further predicts that the mean ratings for standard-accept will be greater than mean ratings for the other 3 treatments. The fourth model is a discretionary model in which neither factor plays a significant role in a layperson’s liability determinations.

Our hypothesis is that lay judgments of liability are driven by both whether the AI recommended the treatment and whether the treatment is standard. If the hypothesis is true, we expect to find the follow-both pattern of results, given the experimental design (8).

The statistical test selected for our primary preregistered hypothesis was a 2 × 2 ANOVA. For the additional preregistered hypotheses, we used 2-sided *t* tests to test for a significant difference and two 1-sided *t* tests to test for equivalence between the 2 conditions in which the 2 influential factors were in conflict (nonstandard-reject and nonstandard-accept). In the case of the two 1-sided *t* tests, we prespecified a medium effect size of 0.5. In all cases, the a priori significance level was 0.05. Unless otherwise noted, analyses were conducted with Stata 15.1MP, which is developed by StataCorp. Each of us participated in the data analysis, as well as Dr. Henry Kim, who provided technical research assistance.

### Procedure

**FIGURE 3.**
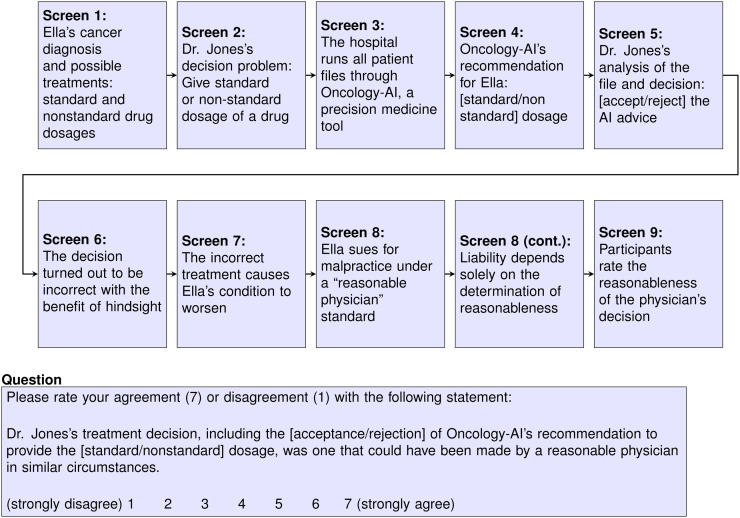
Flowchart of vignette.

The full experimental protocols were previously published (8) and are summarized in the Supplemental Appendix, section I (supplemental materials are available at http://jnm.snmjournals.org) (9,10). A schematic showing the overall flow of the survey scenario text (vignettes) is in Figure 3.

Participants were randomly assigned to 1 scenario (Fig. 1) and evaluated the reasonableness of the physician’s decision on a Likert scale from 1 to 7: was the physician’s treatment decision one that could have been made by a reasonable physician in similar circumstances. Higher scores indicated greater reasonableness and thus lower liability.

The experiment was run with approval from the ETH Zürich Institutional Review Board, and all participants provided written informed consent before participating in the experiment.

## RESULTS

The study, exclusion criteria, analyses, and hypotheses were preregistered with AsPredicted, and all preregistered analyses are reported. The initial response rate (responses to Lucid emails) was 97%; the conversation rate (finishing the study) was 78%. Two thousand sixty participants completed the study; 693 were excluded for failing preregistered comprehension checks, and 11 were excluded for other reasons (e.g., taking the survey twice; Supplemental Appendix, section II). The results are robust to analyzing the data without exclusions (8).

**FIGURE 4.**
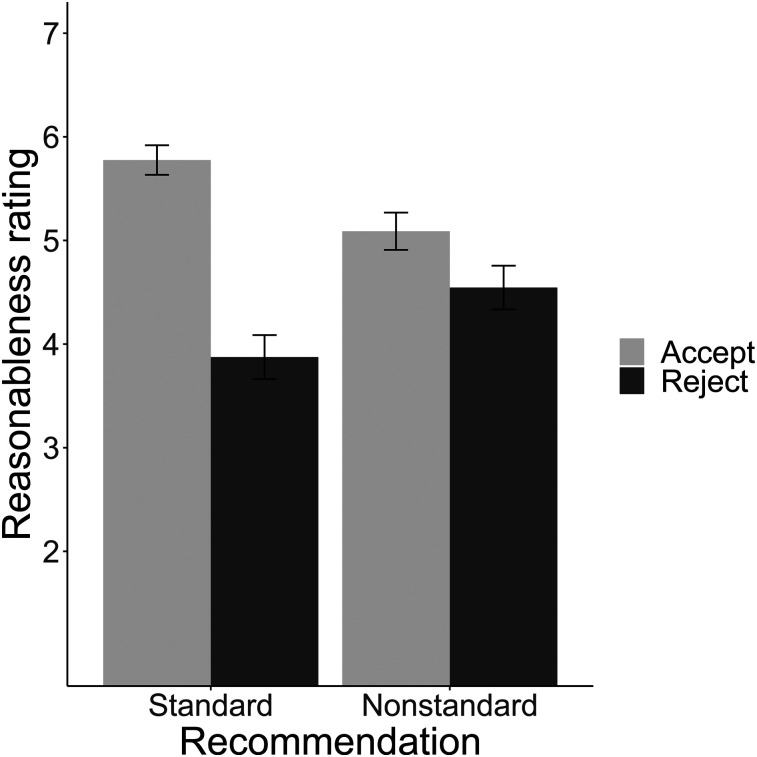
Mean ratings of reasonableness, by condition. Error bars indicate 95% CIs.

We conducted a 2 (recommendation: standard care, nonstandard care) × 2 (decision: accept, reject) ANOVA, treating reasonableness ratings as the dependent variable. As predicted, there was a main effect of decision (*F*_1,1352_ = 167.71, *P* < 0.0001, partial η^2^ = 0.11) and a significant decision × recommendation interaction (*F*_1,1352_ = 51.68, *P* < 0.0001, partial η^2^ = 0.037). There was no significant effect of recommendation (*F*_1,1352_ < 1, *P* = 0.95) (Fig. 4). Ratings were highest for standard-accept (*M* = 5.77; 95% CI, 5.60–5.95) and then were consecutively lower for nonstandard-accept (*M* = 5.09; 95% CI, 4.91–5.27), nonstandard-reject (*M* = 4.55; 95% CI, 4.35–4.75), and finally standard-reject (*M* = 3.87; 95% CI, 3.68–4.07). Table 1 presents pairwise comparisons among conditions.

**TABLE 1:** Pairwise *t* Tests

				*t*		
Scenario	*n*	Mean	SD	Standard-reject	Nonstandard-accept	Nonstandard-reject
Standard-accept	401	5.77	1.46	15.03*, Cohen d = 1.14 [0.97, 1.30]	5.89*, Cohen d = 0.43 [0.28, 0.57]	9.80*, Cohen d = 0.76 [0.60, 0.92]
Standard-reject	311	3.87	1.91		−8.60*, Cohen d = −0.67 [−0.83, −0.51]	−4.39*, Cohen d = −0.36 [−0.52, −0.20]
Nonstandard-accept	360	5.09	1.74			3.86^†^, Cohen d = 0.31 [0.15, 0.46]
Nonstandard-reject	284	4.55	1.81			

* *P* < 0.001.

† *P* < 0.0001.

The 2 post hoc comparisons (standard-reject to nonstandard-accept and nonstandard-reject) reflect Bonferroni-corrected *P* values for 2 post hoc tests. Data in brackets are 95% CIs.

Following our preregistration plan, we next evaluated several pairwise comparisons of interest (Table 1 reports statistics). The mean reasonableness rating in standard-accept was significantly higher than that in standard-reject. It was also significantly higher in standard-accept than in either of the nonstandard conditions. We predicted that the difference between nonstandard-accept and nonstandard-reject would be small (less than Cohen d = 0.5). The analysis was consistent with the equivalence hypothesis with a preregistered medium-sized effect (Cohen d = 0.5; *t*(596) *=* −2.46; *P =* 0.007; 90% CI, 0.31–0.77). Ratings were significantly higher for nonstandard-accept than for nonstandard-reject, but the size of this effect was small. All pairwise *t* tests were to test preregistered hypotheses, except for the comparisons of the standard-reject mean to the nonstandard-accept and nonstandard-reject means, which are 2 post hoc comparisons.

The overall pattern of these results is most consistent with the follow-both model, and we take this to suggest that lay jurors rely on both factors (both AI recommendation and provision of standard care). However, one might wonder whether this pattern is a composite of the follow-AI and follow-standard-care models. Perhaps some participants think that physicians ought to follow only the AI recommendation, whereas others think that physicians should act only according to what is considered standard care in reaching their liability assessment. Under such a heterogeneous-types hypothesis, the follow-both pattern reflects a mixture of those types in the subject pool.

However, the data do not support this heterogeneous-types view. Across all 4 experimental conditions, distributions of the reasonableness ratings were unimodal and not bimodal (8). The heterogenous-types view would predict bimodal distributions when the 2 AI and standard-care factors diverge (e.g., in nonstandard-accept). Moreover, in a series of preregistered follow-up questions, participants ranked how important the follow-AI-recommendation factor and the providing-standard-care factor were to their reasonableness assessment. Most (77%) rated the importance of both factors at or above the midpoint (8).

In addition to these main results, which reflect the analyses in the preregistration, we also collected exploratory data on several other factors, including demographic data (the full battery was previously published (8)). The main ANOVA findings (Fig. 3) are robust to including age, race, and sex as covariates (Supplemental Appendix, section III, Table A1).

## DISCUSSION

Overall, the results strongly support the follow-both model of lay liability judgment. People apply 2 different factors in evaluating physicians who use medical AI systems: whether the treatment provided was standard and whether the physician followed the AI recommendation.

These results have important implications for physicians who seek to minimize tort liability. If physicians receive a standard-care AI recommendation, there is a legal incentive to accept it. All else being equal, participants judge accepting a standard-care recommendation as more reasonable than rejecting it. On the other hand, if physicians receive a nonstandard AI recommendation, they do not necessarily make themselves safer from liability by rejecting it.

Given that physicians who receive nonstandard advice are worse off in terms of liability than physicians who accept standard advice, health-care institutions might consider whether to make AI systems available to physicians. However, the experimental scenarios studied here assume that an AI recommendation is already routinely offered. The study has nothing to say about the relative likelihood of liability for physicians who have not received advice from an AI system and therefore does not support any inference that health-care institutions should avoid introducing AI systems. Additionally, those decisions will likely involve nonlegal factors as well, such as the competitive pressure to maintain state-of-the-art facilities and their ability to set guidelines for the appropriate use of the AI system.

The study most directly examines laypeople as potential jurors, but it also sheds light on laypeople as potential patients. Important recent work in psychology shows that laypeople are algorithm-averse in other forecasting contexts, particularly when they see algorithms err (11). But this study’s results suggest that laypeople are not as strongly averse to physicians’ acceptance of precision medicine recommendations from an AI tool, even when the AI errs. The 2 scenarios rated most reasonable were also those in which the algorithm’s diagnostic advice was wrong for the patient (standard-accept and nonstandard-accept). In the other 2 scenarios (standard-reject and nonstandard-reject), the physician rejected correct AI advice, and this decision was evaluated as more unreasonable. Together, these results suggest that patients may not exhibit strong algorithm aversion in such medical contexts.

Finally, the findings also speak to recent concerns about legal impediments to the use of AI precision medicine (1). Tort law may not impose as great a barrier to the uptake of AI medical system recommendations as is commonly assumed; in fact, it might even encourage the uptake of AI recommendations.

Moreover, we find the same decision effect and decision × recommendation effect on ratings of whether most physicians in similar circumstances would have made the same treatment decision (Supplemental Appendix; section IV) (12). This finding suggests that the results extend to medical negligence standards focused more squarely on judgment of what care is common or customary. More broadly, these exploratory findings are consistent with prior research indicating that lay conceptions of reasonableness are affected by what seems common (Supplemental Appendix, section IV) (12). If this is right, we would predict that as AI use becomes more common, any tort law incentive to accept AI recommendations will only strengthen further. And we would predict this effect both for medical negligence standards centered on custom and those expressed more broadly in terms of reasonableness.

This study had some limitations. It concerned judgments of liability, given that harm occurred. In practice, liability risk is determined by 2 factors: the probability of liability given that harm occurred (factor A, the focus of our study) and the probability of harm occurring at all (factor B). Factor A addresses whether tort law may be a barrier to AI use in medicine. Factor B, however, is best estimated by medical experts with rich knowledge of the specific context. For example, there could be a medical context in which a physician receives AI advice to provide standard care, but the physician is extremely confident that this is the wrong advice and that accepting it will harm the patient. Our findings suggest that the physician would have a degree of protection from the tort law system, which favors providing standard advice and following the AI’s recommendation (factor A). However, if the probability of harm is sufficiently great (factor B), it could—and should—outweigh the tort law incentive to accept standard advice.

There are also limitations in the degree to which the study modeled the procedural elements of a real jury trial. For example, jurors would likely be presented with expert testimony concerning the use of AI precision medicine. Of course, jurors would normally hear expert testimony from each side: one expert that favored taking AI advice and one that disfavored it. Future work could assess whether there is any systematic effect of dueling expert testimony in these cases (e.g., perhaps laypeople generally tend to defer to pro-AI experts) and whether other procedural aspects of a trial complicate the more basic model of lay judgment discovered and presented here.

## CONCLUSION

This study provides—for, what is to our knowledge, the first time—experimental evidence about physicians’ potential liability for using AI in precision medicine. We find that 2 factors reduce lay judgment of liability: following standard care and following the recommendation of AI tools. These results provide guidance to physicians who seek to reduce liability, as well as a response to recent concerns that the risk of liability in tort law may slow the use of AI in precision medicine. Contrary to the predictions of those legal theories, the experiments suggest that the view of the jury pool is surprisingly favorable to the use of AI in precision medicine.

## DISCLOSURE

This study was funded by ETH Zurich and the ETH Fellows Program. No other potential conflict of interest relevant to this article was reported.

**KEY POINTS**

**QUESTION:** How can physicians minimize liability risk when using AI systems?
**PERTINENT FINDINGS:** A representative experimental study of 2,000 U.S. adults found that 2 factors affect liability assessments: whether standard, rather than nonstandard, care was provided and whether the AI advice was accepted.
**IMPLICATIONS FOR PATIENT CARE:** Accepting AI advice reduces physician liability, offering a legal benefit even when there is a nonstandard-care AI recommendation (all else being equal).
